# “Are We in Sync with Each Other?” Exploring the Effects of Cosleeping on Heterosexual Couples' Sleep Using Simultaneous Polysomnography: A Pilot Study

**DOI:** 10.1155/2017/8140672

**Published:** 2017-03-30

**Authors:** Henning Johannes Drews, Sebastian Wallot, Sara Lena Weinhold, Panagiotis Mitkidis, Paul Christian Baier, Andreas Roepstorff, Robert Göder

**Affiliations:** ^1^Department of Psychiatry and Psychotherapy, Christian-Albrechts-University Kiel, Niemannsweg 147, 24105 Kiel, Germany; ^2^Department of Language and Literature, Max Planck Institute for Empirical Aesthetics, Grüneburgweg 14, 60322 Frankfurt am Main, Germany; ^3^Department of Management, Aarhus University, 8000 Aarhus C, Denmark; ^4^Center for Advanced Hindsight, Duke University, Durham, NC, USA; ^5^Interacting Minds Centre, Aarhus University, 8000 Aarhus C, Denmark

## Abstract

The present study aimed to explore dynamic and interactive aspects of cosleep in heterosexual couples. The sample consisted of eight young healthy adults who belonged to four heterosexual couples with a good relationship quality and a history of cosleeping. All individuals underwent simultaneous polysomnography in a sleep laboratory for four nights in which they slept individually and with their partner. Also, a sleep protocol of subjective sleep measures was completed. Statistical analyses included cross recurrence quantification analysis to assess synchronization during sleep. Cosleeping was associated with better subjective sleep quality, increased total sleep time, sleep efficiency, total slow wave sleep, and REM sleep. Sleep stages were more synchronized during cosleep independent of awakenings. Cardiorespiratory measures remained unchanged. The results indicate that young healthy couples in good relationships benefit from cosleeping on a subjective and objective level. Combining simultaneous polysomnography and cross recurrence quantification analysis is a promising method to study dynamic and interactive aspects of cosleep possibly leading to deeper understanding of the role of sleep for sociality, the nature of REM sleep, and the partner as a social zeitgeber. Moreover, clinical implications may arise from these findings.

## 1. Introduction

More than 50% of the adult population in the US share their beds with a significant other [1]. Despite the high prevalence of cosleep, polysomnographic studies on cosleeping couples are rare. Also, the existing polysomnographic studies either look exclusively at static sleep measures [2–5] or monitor one partner only [6]. Thus, they neglect the dynamic and interactive aspects of cosleep, namely, the interpersonal synchronization of sleep architecture or cardiorespiratory physiology.

Understanding cosleep of couples more deeply and addressing its interactive dimension seem important for two reasons. First, cosleep in adults is likely to be a possible source of error in sleep lab diagnostics as it has been shown to differ significantly from sleeping alone (e.g., [3]; for reviews on cosleeping couples see [7, 8]). Moreover, Edinger et al. [6] demonstrated that total sleep time in usually cosleeping insomniacs is systematically underestimated during sleep laboratory diagnostics. Second, synchronization of physiological parameters (i.e., heart rate) is a feature of social interaction in wake people which has attracted attention recently as it varies with sociopsychological relationship characteristics such as social proximity [9] and trust [10]. Interpersonal coupling of cardiorespiratory physiology or sleep stages during cosleep in adults has not been studied before despite possibly providing important insights into social, emotional, and relational aspects of sleep as well as new therapeutic approaches (e.g., partner-based behavioral therapy of chronic insomnia).

Here, we present an explorative pilot study which combines for the first time simultaneous polysomnography, analysis of established sleep measures, and cross recurrence quantification analysis to address the unique features of cosleep in couples.

## 2. Material and Methods

The sample consisted of 8 healthy German natives (mean age 24, ranging from 20 to 29) who belonged to four heterosexual couples. All couples had had coslept prior to study initiation for at least 6 months and were good sleepers without subjective daytime sleepiness as assessed by the Pittsburg Sleep Quality Inventory and the Epworth Sleepiness Scale. In addition, all couples reported a good relationship quality as assessed by the Quality of Relationship Inventory. Relationship quality was controlled for as it is has been reported to impact sleep quality in couples [7]. Moreover, marital satisfaction has been linked to sleep-wake synchrony in married couples, as shown by a recent actigraphic study by Gunn et al. [11].

Each couple slept in our sleep laboratory for two sets of two consecutive nights. The order of sleep modes (individual sleep in separate rooms and cosleep in juxtaposed single beds) was counterbalanced, so that all couples slept in both sleep modes. During each night, both subjects underwent standard polysomnography according to the recommendations of the American Academy of Sleep Medicine [12]. Additionally, the polysomnographs were strictly synchronized. After each night, a protocol of subjective sleep measures was completed. The study design was approved by the ethics committee of Kiel University. Written informed consent was obtained.

Standard sleep measures and protocol data of all nights were analyzed using paired, two-tailed* t*-tests (single sleep versus cosleep). Synchrony between partners was assessed using cross recurrence quantification analysis, a nonlinear correlation technique which is robust concerning nonstationary data, such as prolonged physiological measurements. It is useful for quantifying the correlation between categorical time series [13], such as sleep stages, and physiological signals, that is, heart rate [9].

## 3. Results

**Figure 1:**
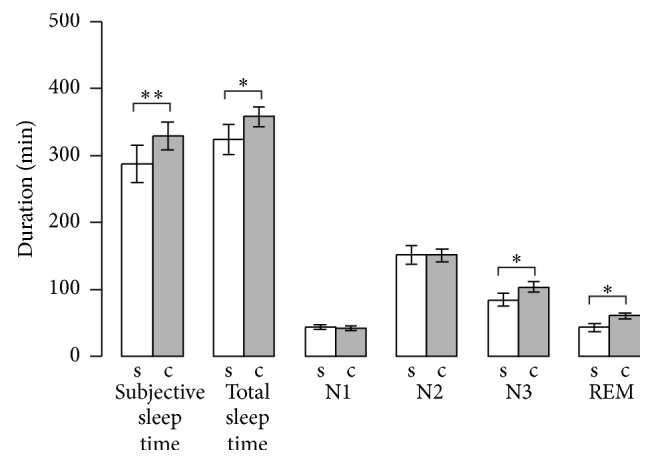
Duration of subjective and objective total sleep time and sleep stages. Mean ± SEM of single sleep (s) and cosleep (c) analyzed using paired, two-tailed* t*-tests. ^*∗*^*P* < 0.05; ^*∗∗*^*P* < 0.01.

Morning protocols showed significantly increased subjective sleep duration when cosleeping (Table 1 and Figure 1). Also, the subjects felt significantly more relaxed after having slept with a partner (Table 1).

When cosleeping, objective total sleep time and sleep efficiency increased significantly, as did total N3 and REM sleep duration and REM sleep proportion (Table 1 and Figure 1). N2 and N3 latencies decreased significantly (Table 1). Standard cardiorespiratory parameters (heart rate, heart rate variation, pulse transit time, pulse transit time decrease, pulse transit time decrease index, and respiratory rate) showed no significant changes (all *P*> 0.187).

**Figure 2:**
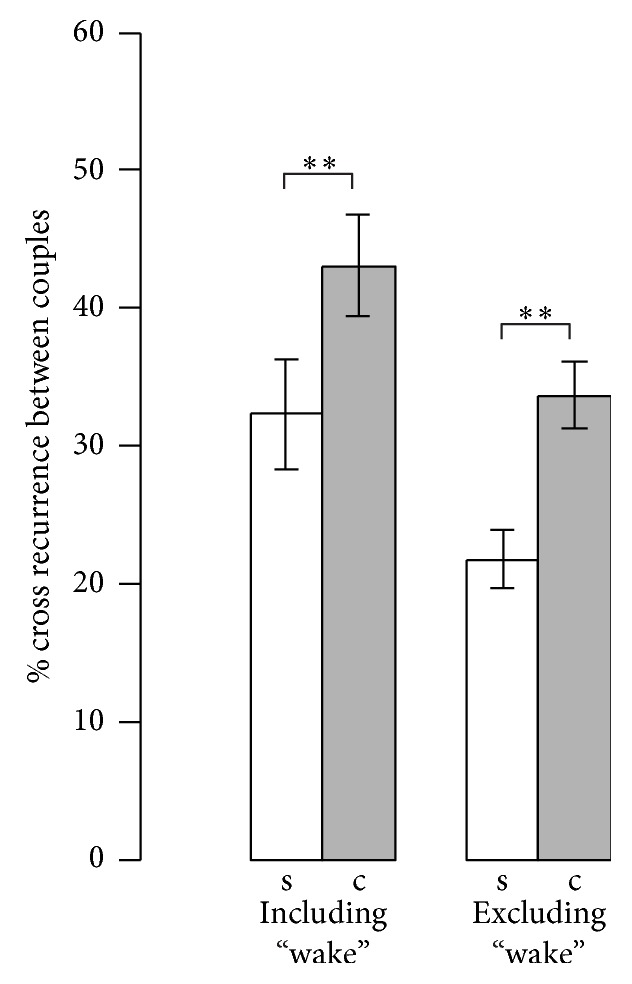
Synchronization of sleep stages. Mean ± SEM of single sleep (s) and cosleep (c) analyzed using paired, two-tailed* t*-tests.^*∗*^*P* < 0.05; ^*∗∗*^*P* < 0.01.

Synchronization of sleep stages was significantly higher under the cosleeping condition. This also holds true if wake episodes are excluded (see Figure 2). However, synchrony in cardiorespiratory physiology (respiratory rate, heart rate) did not change (all *P*> 0.086).

## 4. Discussion

The current study is unique in combining simultaneous polysomnography with analysis of established sleep measures and measures of interpersonal physiological coupling. Our results suggest an improvement in subjective and objective sleep measures as well as increased synchrony of sleep stages during cosleep in young, healthy heterosexual couples in a good relationship as compared to sleeping alone.

### 4.1. Subjective and Objective Measures

Previous studies yield inconsistent results concerning the perceived and measured effect of cosleep on couples. Our findings are in line with studies showing better subjective sleep quality and an increase in total sleep time [3, 14] supporting the notion of cosleep being beneficial on a subjective and objective level.

### 4.2. Changes in Sleep Architecture

Increased total sleep time during cosleep as detected in our study has been described previously in men [14]. As far as sleep stages are concerned, Monroe [3] reports an increase in REM sleep. In our study, similar changes in REM sleep are present across absolute and relative measures. Moreover, a study of Butt et al. [2] using home-polysomnography shows a marked influence of cosleep on REM sleep of younger males.

Thus, the repeatedly reported increase of REM sleep during cosleep might point to a connection of REM sleep to either social or emotional aspects of cosleep which is supported by previous findings showing an increased activity of brain circuits being involved in processing emotional and social information during REM sleep [15].

Regarding changes in slow wave sleep, the aforementioned work by Monroe [3] shows a decrease in N4 sleep. However, given the different sleep stage classification it seems hardly comparable to the present study. In contrast, Butt et al. [16] report a positive correlation of real life social interaction and N3 sleep during the following night in 10 healthy, young married couples. Even though the sleep mode is not explicitly reported in that study and it does not compare cosleep to individual sleep, it nevertheless suggests a connection between N3 sleep and sociality.

### 4.3. Synchrony in Sleep Architecture and Physiology

In sleep research, synchronization between humans has been mainly studied regarding convergence of sleep-wake patterns. Actigraphic studies show a close interdependence of couples' sleep [17], being in line with the notion of partners as social zeitgebers [18]. Also, a concordance of movements during sleep has been stated by a number of works using actigraphy [14, 19, 20]. However, even when employing advanced statistical analyses as presented by Meadows et al. [19] actigraphy only allows for differentiating between wakefulness and sleep. Thus, it remains unclear if the synchronization of movements of cosleeping partners represents a mutual triggering of arousals, disturbing one another's sleep, or whether synchronized movements mirror convergence of otherwise intact sleep cycles. Our findings indicate a zeitgeber effect of a bed partner beyond mere sleep-wake patterning, influencing sleep architecture itself.

Cardiac synchrony in wake people is related to their emotional proximity [9] and trust [10]. In our study, no significant changes in cardiorespiratory synchrony during sleep were observed, representing an interesting difference between wakefulness and sleep to be elucidated in the future.

### 4.4. Possible Clinical Implications

Our study shows significant differences in several sleep parameters between single and cosleep. In addition to their contribution to understanding cosleep in young healthy adults in good relationships, our findings might bear clinical relevance. First, the difference in total sleep time and sleep efficiency supports the notion that sleep lab diagnostics are associated with an underestimation of sleep time in usually cosleeping people, as has been shown in insomniacs by Edinger et al. [6]. Second, the decrease in REM sleep during single sleep might weaken diagnostic accuracy in diseases with high relevance of REM sleep such as REM sleep behavior disorder or REM-related obstructive sleep apnea. Third, the social zeitgeber effect during sleep may indicate beneficial effects of a bed partner in several sleep disorders (i.e., insomnia or circadian rhythm disorders). In insomnia, for instance, it is common to conceptualize a bed partner as a disease promoting factor [21]. Our study shows that cosleeping can be beneficial not only on a subjective level, but also to objective sleep physiology. This might support emerging publications arguing for a prominent role of a bed partner in behavioral therapy for insomnia [21, 22].

However, it is important to note that our sample consisted of healthy couples with a good relationship quality. Thus, the results might not be applicable to couples in conflictual relationships or with one (or both) suffering from a sleep disorder. This of course limits the robustness of the therapeutic implications derived from the present study. For instance, a worsening in relationship quality has been reported to deteriorate sleep in couples in both established sleep parameters [7] and sleep-wake synchrony [11]. Thus, a good relationship quality might be a prerequisite for utilizing the benefits of cosleeping in partner-based therapy.

As far as couples with sleep disorders are concerned, there are indications that cosleeping is beneficial to the sleep-disordered partner [6] and might not disturb the sleep of the healthy sleeper [5]. On the other hand, the healthy individual's sleep has also been reported to be disturbed by the sleep-disordered partner objectively [4] or at least subjectively [23]. Thus, one of the core questions for future research seems to be to determine under which circumstances the beneficial effects of cosleeping for the sleep-disordered individual outweigh the detrimental effects for the healthy cosleeper.

### 4.5. Limitations of the Study

Besides the aforementioned limitations concerning the clinical implications it is noteworthy that the present study is of explorative character. Hence, it is limited in sample size and statistical rigidity; that is, a normal distribution was assumed for the obtained data and no correction for multiple testing was employed. That approach seems justifiable as it was designed to cover a broad and understudied field as is cosleep of couples and thereby avoiding rejecting possible effects prematurely. Also, the study design precludes subgroup analyses such as studying sex/gender effects, which have been suggested to be relevant in cosleep of couples [2, 8].

Moreover, the present study uses a homogeneous sample of young, healthy German natives in a good relationship. Thus, our results might not be applicable to differently composed samples.

## 5. Conclusion and Prospects

This pilot study successfully introduces the combination of simultaneous polysomnography and cross recurrence quantification analysis to study interactive and dynamic aspects of cosleep. In young healthy couples, cosleep differs from individual sleep across subjective sleep measures, sleep architecture (i.e., increased REM sleep and N3 sleep), and sleep stage synchrony, which indicates beneficial effects of cosleep at least in the studied population. The findings need to be reproduced in a bigger and more heterogeneous sample allowing for subgroup analysis in order to study age and gender effects as well as psychosocial and cultural aspects. Future studies should also aim to better understand cosleep in couples with one (or both) suffering from a sleep disorder to further elucidate diagnostic and therapeutic relevance of cosleeping in couples.

## Figures and Tables

**Table 1:** Subjective and objective sleep measures during single sleep and cosleep.

	Mean		SD		*P*
	Single	Couple	Single	Couple	
*Sleep protocols*					
Subjective sleep time (min)	287.5	341.1	113.0	82.1	**0.007**
Feeling recovered (from not at all (1) to very (5))	2.6	2.9	0.8	0.6	0.054
Morning condition (from depressed (1) to lighthearted (6))	3.8	4.1	1.5	1.5	0.232
Morning condition (from feeling run down (1) to refreshed (6))	3.1	3.1	1.2	1.1	0.806
Morning condition (from tense (1) to relaxed (6))	3.5	4.3	1.4	1.5	**0.029**

*PSG-data*					
Total sleep time (min)	324.5	358.0	90.1	57.5	**0.014**
Sleep efficiency (%)	75.1	83.7	19.2	11.3	**0.004**
N1-latency (min)	21.5	22.7	9.8	10.7	0.734
N2-latency (min)	39.7	29.2	21.8	11.0	**0.034**
N3-latency (min)	52.7	41.4	24.1	13.7	**0.045**
REM-latency (min)	147.6	113.6	93.0	64.1	0.113
N1-percentage (%)	16.1	12.6	11.0	6.3	0.083
N2-percentage (%)	45.9	41.8	11.0	6.3	0.077
N3-percentage (%)	25.8	28.6	8.8	7.3	0.164
REM-percentage (%)	12.3	17.0	5.7	3.3	**0.024**
Number of awakenings (*n*)	21.8	22.0	6.3	5.7	0.868
Number of awakenings/hour	4.6	3.8	2.4	1.3	0.087

*Note. P* values < 0.05 are in boldface; *P* = *P* value of paired, two-tailed *t*-tests (single sleep versus cosleep).
